# An *in vitro* cyclic fatigue resistance comparison of conventional and new generation nickel-titanium rotary files

**DOI:** 10.4317/jced.55091

**Published:** 2018-08-01

**Authors:** Celia Ruiz-Sánchez, Vicente Faus-Matoses, Teresa Alegre-Domingo, Ignacio Faus-Matoses, Vicente-José Faus-Llácer

**Affiliations:** 1 Master of Restorative Dentistry and Endodontics, Department of Stomatology, Medicine and Dental School, Valencia University, Spain; 2 DDS, MSc, PhD. Co-director of the Master of Restorative Dentistry and Endodontics, Department of Stomatology, Medicine and Dental School, Valencia University, Spain; 3 DDS, MSc, PhD. Professor of the Master of Restorative Dentistry and Endodontics, Department of Stomatology, Medicine and Dental School, Valencia University, Spain; 4 DDS, MSc, PhD. Professor of the Master in Orthodontics, Department of Stomatology, Medicine and Dental School, Valencia University, Spain; 5 MD, DDS, PhD. Director of the Master of Restorative Dentistry and Endodontics, Department of Stomatology, Medicine and Dental School, Valencia University, Spain

## Abstract

**Background:**

New designs and processing of Niquel-Titanium (NiTi) have been introduced to increase resistance to cyclic fatigue. The purpose of this study was to compare the cyclic fatigue resistance of 3 NiTi rotary instruments, ProTaper Next (PTN; Dentsply Maillefer, Ballaigues, Switzerland), Profile Vortex Blue (PVB; Dentsply Tulsa Dental, Tulsa, OK, USA) and ProTaper Universal (PTU; Dentsply Maillefer, Ballaigues, Switzerland).

**Material and Methods:**

A cyclic fatigue test was conducted operating instruments from ProTaper Next X2, Profile Vortex Blue 25.06 and ProTaper F2. A total of 234 instruments were rotated in 2 simulated stainless steel curved canals with different angles of curvature (45º and 60°) and 5-mm radius of curvature. The number of cycles to fracture (NCF) was calculated. Data were compared using 2-way analysis of variance and post-hoc Bonferroni test in software (SPSS 15.0, Chicago, IL). Statistical significance was set at *P*<0.05.

**Results:**

Profile Vortex Blue showed higher resistance to cyclic fatigue in both curved canals than ProTaper Next and ProTaper Universal (*P*<0.001). ProTaper Universal obtained the lowest resistance to cyclic fatigue in both canals (*P*<0.001).

**Conclusions:**

Profile Vortex Blue was the most resistant to cyclic fatigue failure, followed by ProTaper Next and ProTaper Universal. Anatomical complexity (angle of curvature) and manufacturing process of NiTi are important factors for resistance to cyclic fatigue.

** Key words:**Cyclic fatigue, M-Wire, Protaper Next, ProTaper Universal, Profile Vortex Blue.

## Introduction

Unexpected fracture of rotary systems remains a major concern for clinic, despite improvements in NiTi alloy (1,2). Clinical fracture of NiTi instruments incidence ranges between 0.26%-21% (2-4). The main reason for these fractures is the cyclic fatigue (5,6).

Advances in technology and manufacturing process of NiTi alloy have resulted in a new generation of files with superior physical-mechanical properties, flexibility and resistance to cyclic fatigue (7,8). Different designs, alloys and manufacturing methods have been proposed in order to reduce fractures (9). It is the case of M-Wire NiTi alloy, developed through thermo-mechanical processing applied to Nitinol 508 (10-12). ProTaper Next (PTN; Dentsply Maillefer, Ballaigues, Switzerland) are made of this NiTi M-Wire alloy. They have been proven better properties of NiTi M-Wire compared with conventional NiTi (10,13-16). By contrast, the rotary system ProTaper Universal has the conventional NiTi alloy. Comparing ProTaper Next system in a cyclic fatigue test against ProTaper Universal, NCF was higher (13,17).

Manufacturers have improved fracture resistance by eliminating surface irregularities (machining marks) and applying various heat treatments to NiTi alloy (18). Current research focuses on improving the surface of NiTi instruments. The use of surface treatment techniques has been shown to improve flexibility, surface hardness, cut performance and wear resistance of NiTi instruments (19,20). Profile Vortex Blue (PVB; Dentsply Tulsa Dental Specialties, Tulsa, Oklahoma, USA) are manufactured with M-Wire NiTi alloy subjected to an advanced thermo-mechanical process. As a result of this manufacturing process a titanium oxide surface layer is obtained, giving them their characteristic blue colour (11,14). They have probed to show an improvement resistance to cyclic fatigue compared to NiTi M-Wire NiTi and conventional NiTi (14,16,21). The aim of this study was to compare *in vitro* resistance to cyclic fatigue of Profile Vortex Blue, Next and ProTaper ProTaper Universal.

## Material and Methods

Cyclic fatigue test was conducted by operating instruments from ProTaper Next (PTN) X2, Profile Vortex Blue (PVB) 25.06 and ProTaper Universal (PTU) F2. A total of 234 instruments (n=39 per group) were rotated in 2 simulated curved canals. To compare them, the same diameter and length were selected, and a taper as similar as possible.

**Figure 1:**
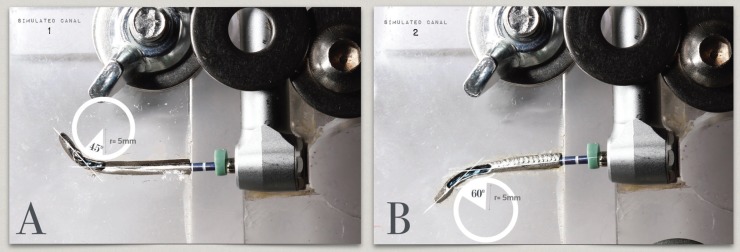
The 2 artificial canals. Canal 1 (A), 45º angle and 5-mm radius; canal 2 (B), 60º angle and 5-mm radius.

The static fatigue test device used in this study was a modification (22,23) from that used in previous published studies (24,25). The device consisted of two stainless steel artificial canals inserted in an area of methacrylate where the handpiece was fixed to eliminate operator pressure bias when performing axial movements. The canal 1 had a curvature angle of 45° (Fig. 1A) and the canal 2 had a curvature angle of 60° (Fig. 1B); both canals had a radius of curvature of 5 mm. Artificial conduits have a length of 20 mm, a tip of 0.40 mm and a taper of 9%. The diameter of the simulated canals was superior to the instruments, allowing free rotation of the file into the canal.

Instruments were rotated in an electric motor (X-Smart, Dentsply Maillefer, Ballaigues, Switzerland) using a conventional rotary motion with a handpiece reduction of 16:01, with a constant speed and torque recommended by the manufacturer of 300 rpm and 5.2 N/cm for PTN and PTU, and 500 rpm and 2.8 N/cm for PVB. The working length was standardized to 19 mm for all files. To reduce the friction of the files with the metal canals walls during conducting the test and minimize the release of heat, lubricant oil (Millet-Franklin, BA, Argentina) was applied within the artificial conduits before each use. All instruments were rotated until fracture occurred. The time until failure was recorded with a camera attached to a tripod (Canon EOS 600D, Canon Incorporated, Tokyo, Japan) and a digital timer (Timex, Middlebury, CT), stopping as soon as the fracture was detected. Time was converted into the number of cycles to fracture (NCF). By converting to number of cycles to fracture, you can make comparisons between files rotated at different speeds (8).

Data were compared with software (SPSS 15.0, Chicago, IL) by 2-way analysis of variance (ANOVA) and post-hoc Bonferroni test. As a control measure, the non-parametric Kruskal-Wallis and Mann-Whitney tests were applied. Statistical significance was set at *P*<0.05.

## Results

**Figure 2:**
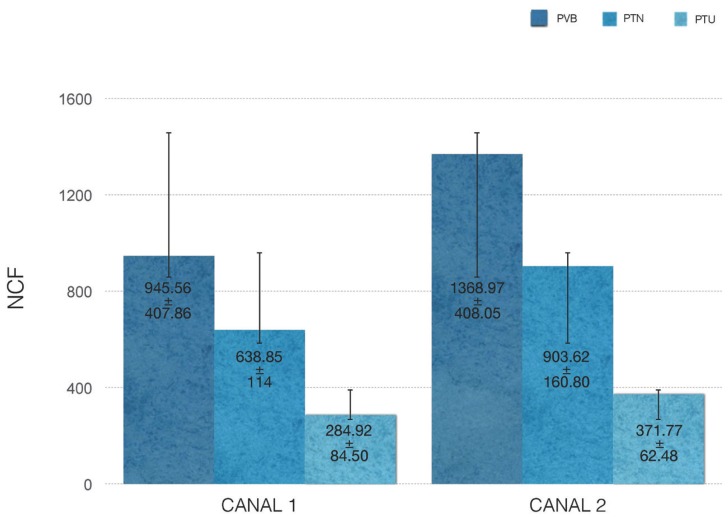
Mean NCF +- standard deviations for each instrument type in each canal. The most difficult curvature (canal 1) generated the least NCF in the 3 systems compared (*P*< .05); the easiest canal (canal 2) showed the highest NCF in all instruments (*P*< .05).

**Figure 3:**
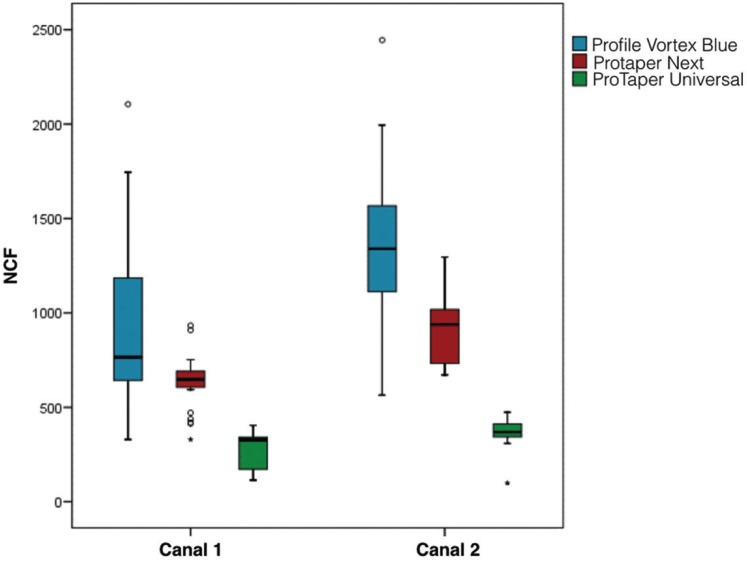
The box-plot graphic allows visualizing the entire distribution of rotations values recorded.

The NCFs for each file in each of the canals were presented in Figure 2 and Figure 3. PVB obtained the highest NCF (*P*<0.001), followed by PTN and PTU (*P*<0.001). Regarding the canal typology, PVB and PTN were significantly higher in the canal 2 than in the canal 1 (*P*<0.001). Into canal 2, it was more evident the superiority of PVB against PTN (*P*<0.001). However, for PTU was not enough statistical evidence that it worked better in a type of canal or other (*P*=0.130). The NCF depends on the angle of curvature, resulting lower values in canals with more anatomical complexity (higher angle of curvature).

## Discussion

Thanks to improvements in instrument design and manufacture of NiTi alloys, clinical can prepare the root canals in a more efficient and faster manner than with stainless steel files (14). However, fracture is still higher compared to manual instruments (1,2). The mechanical behavior of NiTi alloys is sensitive to the microstructure and associated to thermo-mechanical treatment history (4). To improve the fatigue strength should be optimized the microstructure of NiTi alloys through new manufacturing processes and treatment of alloys (9).

During manufacture of NiTi rotary instruments, small scratches and grooves occurred on the surface. When stress is concentrated on these machining marks, microcrack initiation occurs. The propagation of these microcracks is a major cause of unexpected fracture (26,27). Through the use of surface treatment techniques, is attempted to reduce these surface irregularities (18). There are several methods for surface treatment of metal, such as ion implantation, thermal nitration, cryogenic treatment and electro-polishing. From among the most frequently used, electro-polishing and ion implantation have been shown to improve the mechanical properties and finish of the surface of metals, as well as superior resistance to cyclic fatigue (18,28,29).

Three rotary files systems with different processing of NiTi were selected to evaluate the resistance to cyclic fatigue. The results of this study show that the most resistant to cyclic fatigue was Profile Blue Vortex, followed by ProTaper Next and ProTaper Universal. These results coincide with the only similar published study (21). This study compared the complete system of each file (n=20 per group), so our sample size proportion was higher (n=39 per group). Furthermore, they used a single curvature angle of 90°. Because of that it remains difficult to compare the results.

The cyclic fatigue resistance is determined by the properties of the NiTi instruments, such as cross-section design, tip diameter, taper, materials and manufacturing processes (6,11,14,30). Apart from factors such as speed of rotation, the radius and angle of curvature, and the type of continuous or reciprocating motion with which are driven (22,31,32). The combination of this has hindered the comparisons between the three rotary systems analysed.

The geometric of the root canal (radius and angle of curvature) is one of the most important factors in failure of NiTi instruments (6,22,33). In a complex canal anatomy the lowest NCF results were obtained. Hence one can deduce that the angle of curvature influences the resistance to cyclic fatigue, coinciding with the results of other studies (6,22,33).

A higher cross-sectional area was associated with less flexibility and worse cyclic fatigue resistance (30). The rotary system ProTaper Universal has a convex triangular cross-section, ProTaper Next has an offset rectangular and Profile Vortex Blue has a triangular cross-section. Profile Vortex Blue were the most resistant to cyclic fatigue, confirming that the decrease in cross-sectional area has a significant effect on cyclic fatigue resistance (30).
